# A Novel Method to Titrate Herpes Simplex Virus-1 (HSV-1) Using Laser-Based Scanning of Near-Infrared Fluorophores Conjugated Antibodies

**DOI:** 10.3389/fmicb.2017.01085

**Published:** 2017-06-14

**Authors:** Marco Fabiani, Dolores Limongi, Anna Teresa Palamara, Giovanna De Chiara, Maria Elena Marcocci

**Affiliations:** ^1^Department of Public Health and Infectious Diseases, Sapienza University of RomeRome, Italy; ^2^San Raffaele Pisana, Istituto di Ricovero e Cura a Carattere Scientifico, Telematic UniversityRome, Italy; ^3^Department of Public Health and Infectious Diseases, Sapienza University of Rome, Laboratory Affiliated to Istituto Pasteur Italia – Fondazione Cenci BolognettiRome, Italy; ^4^Institute of Translational Pharmacology, National Research CouncilRome, Italy

**Keywords:** HSV-1, herpes simplex virus, plaque assay, virus titration, near-infrared fluorescence, immunostaining, antivirals

## Abstract

Among several strategies used for Herpes simplex virus (HSV) detection in biological specimens, standard plaque assay (SPA) remains the most reliable method to evaluate virus infectivity and quantify viral replication. However, it is a manual procedure, thereby affected by operator subjectivity, and it may be particularly laborious for multiple sample analysis. Here we describe an innovative method to perform the titration of HSV type 1 (HSV-1) in different samples, using the “In-Cell Western^TM^” Assay (ICW) from LI-COR, a quantitative immunofluorescence assay that exploits laser-based scanning of near infrared (NIR). In particular, we employed NIR-immunodetection of viral proteins to monitor foci of HSV-1 infection in cell monolayers, and exploited an automated detection of their fluorescence intensity to evaluate virus titre. This innovative method produced similar and superimposable values compared to SPA, but it is faster and can be performed in 96 well plate, thus allowing to easily and quickly analyze and quantify many samples in parallel. These features make our method particularly suitable for the screening and characterization of antiviral compounds, as we demonstrated by testing acyclovir (ACV), the main anti-HSV-1 drug. Moreover, we developed a new data analysis system that allowed to overcome potential bias due to unspecific florescence signals, thus improving data reproducibility. Overall, our method may represents a useful tool for both clinical and research purposes.

## Introduction

Herpes simplex viruses (HSV) are enveloped DNA-viruses, causing recurrent infections in different sites including lips, eyes (especially for HSV type 1, HSV-1) and genital tract (particularly for HSV type 2, HSV-2). Following a primary infection, they are able to establish a latent infection in neuronal ganglia that is usually followed by lifelong periodic reactivations. They can also reach the central nervous system causing severe forms of encephalitis. During productive infection, HSV efficiently redirect the host transcriptional machinery to express their own genes in a precise regulated temporal cascade, consisting of the sequential expression of three gene classes: the immediate-early (IE, including Infected Cell Protein 4, ICP4), early (E) and late (L, including glycoprotein B, gB) genes (Roizman and Knipe, 2001).

Several strategies are used for HSV detection in biological specimens. Among these, the amplification of viral specific genes through polymerase chain reaction (PCR) (Espy et al., 2000; Van der Beek et al., 2013) allows to check and eventually quantify the viral DNA, but it does not give specific information about the replicative efficiency of the virus and its infectivity.

Alternatively, HSV antigens can be detected by direct immunofluorescence (IF) assay using fluorescein-labeled monoclonal antibodies or enzyme immunoassay (Forghani et al., 1985; Baker et al., 1989; Verano and Michalski, 1990), though their sensitivity is quite low.

So far, the most reliable method to detect and titrate HSV in biological samples remains the virus isolation and quantification by standard plaque assay (SPA). In this method, a confluent monolayer of permissive cells is incubated with the biological sample potentially containing HSV, and then covered with an immobilizing semisolid overlay medium to prevent indiscriminate spreading of neo-formed virions. Thus, viral infection and replication are constrained to the surrounding cells and individual plaques, or zones of cell death, become detectable and countable. The HSV titre results from the number of formed plaques per milliliter of sample, and it is expressed as plaque forming unit per ml (PFU/ml) (Langlois et al., 1986; Kruppenbacher et al., 1994).

Standard plaque assay is also used for basic research purposes, i.e., to check the efficacy of HSV infection in cells by evaluating the titre of neo-formed virion released in cell supernatants.

Plaque reduction assay (PRA), a SPA variant, is exploited to determine HSV susceptibility to antiviral drugs, allowing to calculate the drug concentration able to reduce plaque formation by 50% (IC50) (Safrin et al., 1994). Although SPA is a very sensitive and specific method, it may be particularly laborious and time-consuming for multiple sample analysis and is affected by operator subjectivity in plaque counting.

Here we describe an innovative method we set up to perform HSV-1 titration in different samples, using the “In-Cell Western^TM^” Assay (ICW) from LI-COR, a quantitative IF assay that utilizes laser-based scanning of near infrared (NIR). In particular, we employed viral protein infrared-immunodetection to monitor foci of HSV-1 infection in cell monolayer. This innovative technique produced similar and superimposable values compared to SPA. Moreover, by using ICW to evaluate the IC50 of Acyclovir (ACV), the main anti-HSV-1 drug, we provided evidence demonstrating that this method is suitable for the screening of antiviral compounds, showing several advantages compared to both SPA and PRA. Furthermore, by exploiting an alternative software, we developed a new data analysis method.

## Materials and Methods

### Cells and Viruses

African green monkey kidney (Vero) cells were grown in RPMI 1640 medium supplemented with 10% heat-inactivated fetal bovine serum (FBS), 1% glutamine, 50 U per ml penicillin, and 50 μg/ml streptomycin. All reagents were purchased by Sigma–Aldrich, St. Louis, MO, United States. The cells were maintained at 37°C in humidified air containing 5% CO_2_.

Virus production was performed as previously reported (De Chiara et al., 2016). Briefly, monolayers of Vero cells in 75-cm^2^ tissue culture flasks were infected with HSV-1 strain F (according to appropriate guidelines for the use and handling of BSL-2 pathogenic microorganisms as HSV-1) (Burnett et al., 2009) at a multiplicity of infection (m.o.i.) of 0.01. After 48 h at 37°C, HSV-1 infected cells were harvested with 3 freeze-and-thaw cycles, and cellular debris were removed with low-speed centrifugation, and the supernatant was stored at -80°C, until used. Virus titer in the supernatant was measured by conventional SPA (Killington and Powell, 1991). The titer of the virus preparation was 5 × 10^7^ PFU/ml.

### HSV-1 Staining Using ICW

Vero cells were seeded in 96 well plate and grown to 90% confluency for 24 h. Then, cells were infected with serial dilutions of HSV-1 in RPMI, or supernatants from HSV-1-infected cells. After 1 h of incubation at 37°C, supernatants were aspirated, cells washed two times with PBS, and then incubated with 2% FBS-RPMI for 24 h at 37C° in 5% CO_2_ in the presence or absence of ACV. ACV dilutions were prepared as described (Sarisky et al., 2001; Civitelli et al., 2014). Cells were washed with PBS twice and fixed with 50 μl of 4% paraformaldehyde in PBS for 15 min at room temperature (rt), and then were permeabilized in 0.1% triton X-100 PBS for 5 min at rt. Following the incubation with Odyssey Blocking Buffer for 1 h at rt, cells were incubated with anti-glycoprotein B (gB) or anti-ICP4 antibodies (sc-56987 and sc-S9808N, Santa Cruz, respectively, 1:1000 dilution in Odyssey Blocking buffer) for 1 h and then washed three times with PBS containing 0.1% Tween-20. Afterward, labeled secondary antibody IRDye 800 CW Goat Anti Mouse (926-32210 LI-COR Biosciences, 1:1000 dilution in Odyssey Blocking buffer) and CellTag 700 Stain (926-41090, LI-COR Biosciences, 1:500) were added to each well, and after 1 h, cells were washed four times with PBS containing 0.1% Tween-20. Finally, the plate was scanned on the Odyssey Infrared Imager, and the integrated intensity value of each well read by LI-COR Image Studio Software developed for Odyssey analysis.

### HSV-1 Titre Evaluation by ICW

LI-COR Image Studio Software, developed for Odyssey analysis, was used to acquire the integrated fluorescence intensity from each well. To evaluate virus titre, a standard curve was performed for each experiment by infecting Vero cells with 10-fold serial dilutions of virus stock (stock titre: 5 × 10^7^ PFU/ml), and using mock-infected wells to assess fluorescence intensity background. Standard curves were built by plotting integrated fluorescence intensity (a.u.) measured for each virus dilution versus its relative viral titre expressed as log PFU/ml, and exhibit a sigmoidal or S-shaped trend. HSV-1 titre in unknown samples (i.e., supernatans from HSV-1 infected cells and HSV-1 infection in the presence of ACV) was interpolate from standard curve using the mathematical model four-parameter logistic-log (Sigmoidal 4PL) in GraphPad Prism v6 (GraphPad Software, Inc.).

### HSV-1 Plaque Assay

Different 10-fold dilutions of HSV-1-containing samples were incubated on monolayers of Vero cells (24 well plate) at 37°C for 1 h to allow the virus to attach and entry into cells. Then supernatants were aspirated, cells washed two times with PBS, and incubated with 2% carboxymethyl-cellulose (CMC) in 2% FBS-RPMI for 3 days at 37°C in 5% CO_2_ to allow plaque formation. For plaque counting, the cells were fixed with ice-cold 100% methanol for 20 min at -20°C and stained with 0.5% crystal violet in 10% ethanol for 10 min. HSV-1 titre was calculated through plaque counting and expressed as PFU/ml. All the reagents were purchased from Sigma–Aldrich.

### Data Analysis System

A data analysis system was developed by exploiting a Java-based image processing program (IMAGE-J), that allows to identify the surface area occupied by fluorescence in each well and to calculate from this the output parameter ‘area percentage,’ i.e., the percentage of well area covered by fluorescence (Guzman et al., 2014). For this evaluation, the image obtained by Odyssey Software was converted in a single 8-bit grayscale image for each fluorescence channel. In 8-bit greyscale images there are 256 intensity graduations where the intensity is zero for pixels that do not contain fluorescence and a number between 1 and 255 for pixels containing fluorescence.

By using the command “Image → Adjust → Threshold” specific threshold lines were set for each experiment to select pixels falling within a specified range. In particular, the integrated intensity from uninfected cells was used to set the lowest threshold value, whereas integrated intensity values from unspecific fluorescence peaks were used to set the maximum threshold. Thus, the selected specific signals between these threshold lines were used to calculate the ‘area percentage’ covered by fluorescence for each well. The resulting values were fitted by a non-linear regression using the mathematical model log (inhibitor) vs. normalized response in GraphPad Prism v6 (GraphPad Software, Inc.) to determine ACV IC50.

## Results

### ICW Is a Suitable Technique to Detect HSV-1 Infection in Cells

**FIGURE 1:**
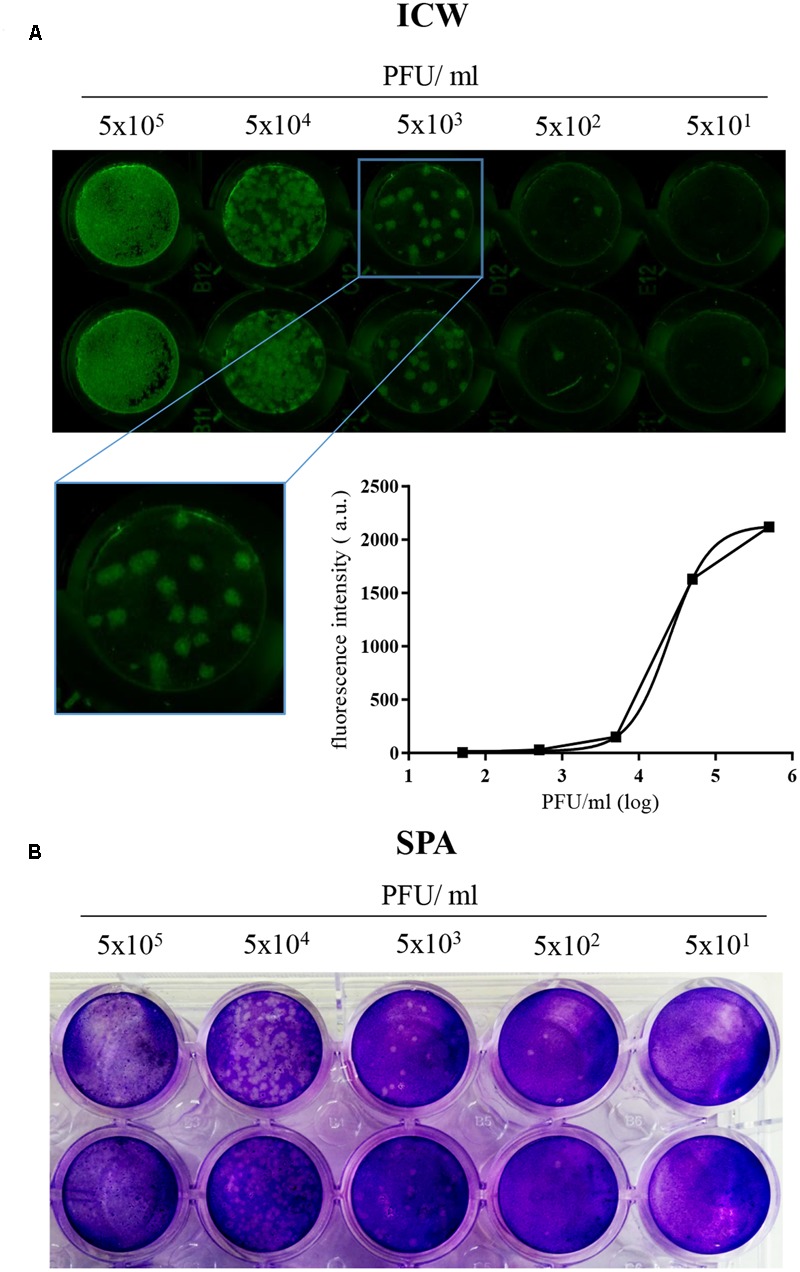
“In-Cell Western^TM^” Assay is a suitable technique to detect HSV-1 infection in cells. **(A)** Vero cells were seeded in 96 wells and infected with the indicated 10-fold serial dilutions of HSV-1. 24 h p.i. cells were fixed and immunostained with anti-gB antibody, followed by incubation with IRDye 800CW goat anti-mouse antibody, and relative fluorescence detected with LI-COR Odyssey Infrared Imaging System. A representative image of one of the three experiments performed is shown, with a close up image of HSV-1 foci of infection (inset) and the relative standard curve built up with the mean value of fluorescence intensity (arbitrary unit, a.u.) detected for each experimental point. **(B)** Representative image of SPA performed with the same HSV-1 dilutions used in **(A)**.

To check whether ICW is a suitable technique to monitor HSV-1 infection, we performed a pilot experiment by infecting Vero cells with serial dilutions of the virus, starting from 5 × 10^5^ PFU/ml. The cells were fixed 24 h p.i. and then stained with anti-gB and with the appropriate NIR-secondary antibody, followed by scanning on the Odyssey Infrared Imager. Mock-infected cells were used as controls to evaluate the threshold of autofluorescence background. In parallel, the same HSV-1 dilutions were also titrated by SPA. Results in **Figure 1A** show that fluorescence intensity from HSV-1-infected cells grew with virus challenge (PFU/ml), indicating that ICW technique could monitor virus infection. Moreover, gB staining resulted in foci of infection, whose morphology (see also inset in panel A) was comparable to those evidenced by SPA (**Figure 1B**), indicating that gB protein expression is a suitable marker to quantify neo-virion production. Thus, we used for the following experiments gB staining to detect/quantify HSV-1 infection.

### ICW Technique vs. Standard Plaque Method

**FIGURE 2:**
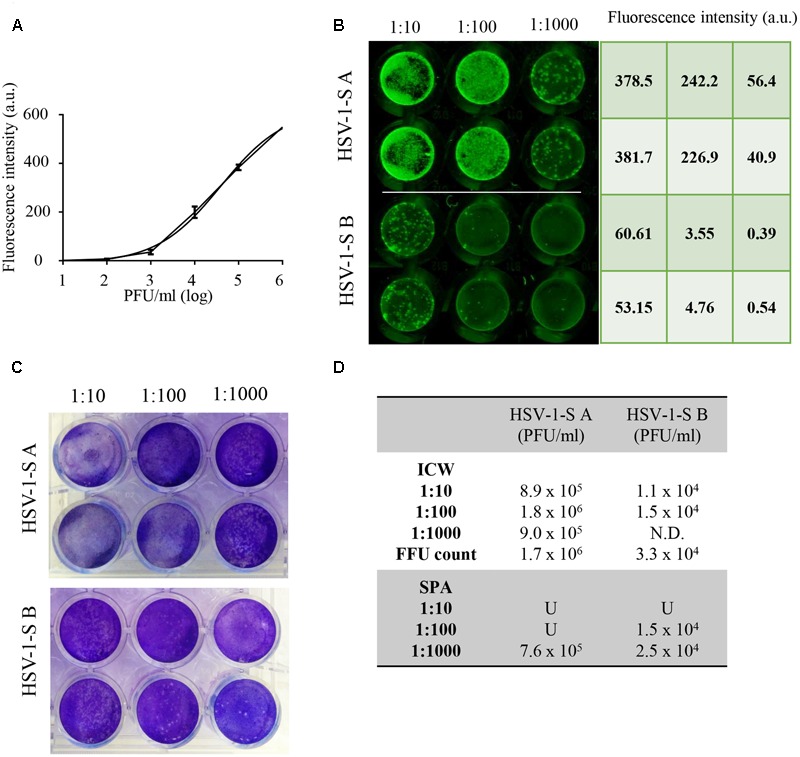
“In-Cell Western^TM^” Assay vs. standard plaque assay. **(A)** ICW Internal standard curve built up by using the mean value of intensity fluorescence detected in Vero cells infected with 10-fold serial dilutions of HSV-1 with a known titre. **(B,C)** Two unknown HSV-1 samples (HSV-1-SA and HSV-1-SB) were titrated using ICW **(B)** and SPA **(C)** as described in Section “Materials and Methods.” **(D)** Mean values of HSV-1-SA and HSV-1-SB titer for each tested dilution in the two assays. N.D. = not detectable; U = uncountable.

To evaluate whether ICW technique may be exploited for HSV-1 titration in biological samples, we used this method in comparison with SPA to quantify virus titre in two supernatants from HSV-1-infected cells (HSV-1-SA and HSV-1-SB). First, serial dilutions of HSV-1 with a known titre were tested by ICW on Vero cells plated in 96 wells to build up the standard curve with the relative fluorescence intensity values (**Figure 2A**). On the same plate, three dilutions of each sample were tested in duplicate (**Figure 2B**) and their HSV-1 titre was calculated by interpolating their fluorescence values on the standard curve, resulting in 1.2 × 10^6^ ± 5.2 × 10^5^PFU/ml (HSV-1-SA) and 1.3 × 10^4^ ± 2.8 × 10^3^ PFU/ml (HSV-1-SB). In parallel, viral titres were measured by SPA (**Figure 2C**) resulting in 7.6 × 10^5^ PFU/ml and 2.0 × 10^4^ ± 7 × 10^3^ PFU/ml for HSV-1SA and HSV-1SB, respectively. Thus, virus titre calculated with ICW was almost completely superimposable to that resulting from SPA (**Figure 2D**). We also evaluated HSV-1 titre by counting the number of gB-detected HSV-1 foci (FFU), obtaining similar results (**Figure 2D**). It is noteworthy that in our hands ICW was more suitable than SPA for these titrations, especially for 1:10/1:100 dilutions that resulted in too high and uncountable number of plaques in SPA. These results demonstrate that ICW technique using “Odyssey^®^ CLx Imaging System” is an accurate and fast method for HSV-1 titration that requires a lower number of cells and sample-dilutions with respect to SPA.

### ICW Technique for Antiviral Drug Screening

**FIGURE 3:**
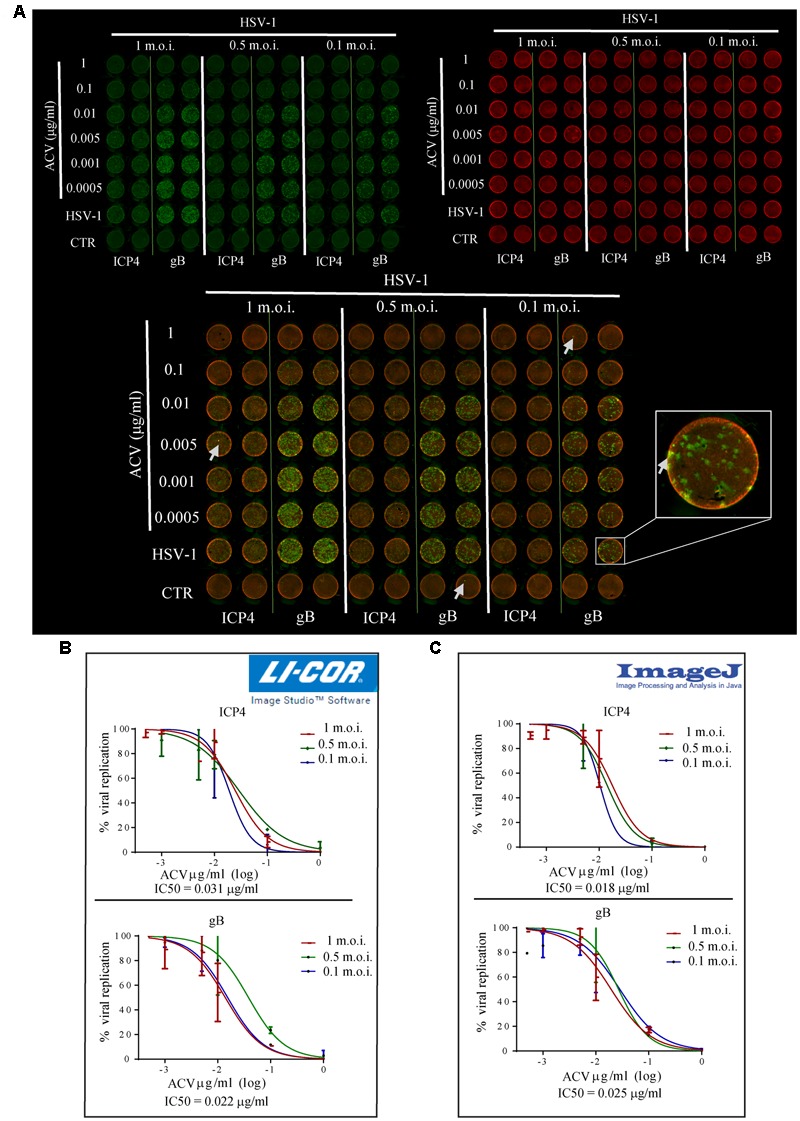
“In-Cell Western^TM^” Assay is a suitable technique for antiviral screening. **(A)** HSV-1 infected Vero cells (with the indicated m.o.i.) were treated for 24 h with concentrations of ACV ranging from 0.0005 to 1 μg/ml. Fixed cells were incubated with anti-gB or anti-ICP4 antibodies and then stained with IRDye 800 CW (green fluorescence) and CellTag 700 Stain (red fluorescence). Arrows highlight unspecific fluorescence peaks (a close up view in the inset). **(B,C)** Representative dose-response curves of ACV activity on HSV-1 replication (shown as percentage of virus replication in ACV treated cells vs. untreated ones) using LI-COR Image Studio Software **(B)** or Image-J **(C)** for data analysis. ACV IC50 was calculated from each staining and the mean values resulted from experiments performed with different m.o.i. of virus are reported (0.031 μg/ml for ICP4 staining, 0.022 μg/ml for gB staining using LI-COR Image Studio Software; 0.018 μg/ml for ICP4 staining, 0.025 μg/ml for gB staining using Image-J software).

To evaluate whether ICW method may be exploited for the screening of anti-HSV-1 drugs, we tested virus titre in Vero cells infected in the presence of ACV, the main inhibitor of HSV-1 replication (Elion, 1982). Vero cells seeded in 96 wells were infected with HSV-1 at different m.o.i. (0.1, 0.5, and 1) and treated with serial concentrations of ACV ranging from 0.0005 to 1 μg/ml. Twenty-four hour later, cells were fixed and stained with antibodies raised against ICP4 or gB, early and late viral proteins, respectively (green fluorescence, **Figure 3A**). In this set of experiments, cells were also stained with Cell-Tag 700, a fluorescent dye that stains cells and allows to detect cell layer (red fluorescence in **Figure 3A**), in order to normalize viral protein fluorescence intensity to cell number. Mock-infected cells were used as controls, and their intensity used as background. Normalized fluorescence intensity resulting from each staining was used to evaluate viral replication. **Figure 3B** shows the percentage (%) of viral replication measured in cells infected in the presence of different ACV concentrations versus those measured in HSV-1-infected cells (100%). ACV IC50 was calculated from each staining (ICP4: upper graph; gB: lower graph), obtaining similar values (0.031 μg/ml for ICP4 staining vs. 0.022 μg/ml for gB staining). The slight discrepancy may be due to unspecific fluorescence peaks observed for each staining (see inset and arrows in **Figure 3A**).

To avoid these random acquisition troubles, we developed a new and innovative data analysis system, by exploiting a Java-based image processing program (IMAGE-J) that allowed to normalize for each well the fraction-area covered by green fluorescence to total area. In particular, unspecific fluorescence peaks were excluded from the selected fraction-area by using specific threshold lines. Normalized fraction area was used to calculate viral replication in each well. The IC50 for ACV was calculated from graphs in **Figure 3C**, resulting in superimposable values (0.018 μg/ml for ICP4 staining, upper graph, 0.025 μg/ml for gB, lower graph). These results were also compared to those obtained through a parallel PRA assay, performed using the same ACV concentrations to treat Vero cells following HSV-1-infection at 0.1 m.o.i.. We found similar results (0.03 < IC50 < 0.07 μg/ml, Supplementary Figure S1).

Overall, these results strongly support ICW as suitable technique for HSV-1 titration in biological samples and antiviral drug screening.

## Discussion

Herein, we describe an innovative method for HSV-1 titration, which allows to easily and quickly analyze viral replication and infectivity in many samples in parallel. Thanks to these features, it is particularly suitable in experimental virology and especially for the screening of antiviral compounds. Moreover, this method shows a greater flexibility than standard SPA, since it can be exploited for different viruses causing or not cytopathic effects (CPE) (Counihan et al., 2006; Weldon et al., 2010; Kumar et al., 2012).

Until now, SPA was considered the most accurate method for the direct quantification of infectious virions, including HSV-1, as well as for screening of antiviral compounds (as PRA). This method is based on the counting of discrete plaques caused by virus-induced CPE, considered a marker of infectious viral units. Unfortunately, it is time consuming (e.g., 48–72 h for HSV-1), especially because virus-induced CPE may take a long time to be visualized as plaques. Moreover, it generally needs high volume of samples (70–700 μl to be analyzed on cell seeded in 24 well plate) and the results may be affected by several technical problems, such those related to the cells susceptibility or to cell overlay systems (Borisevich et al., 2007; Baer and Kehn-Hall, 2014) and potential human bias introduced by manual plaque counting. Recently Tardif et al. (2014) developed a chemiluminescence assay that allows to detect viral CPE indirectly (through measuring ATP-dependent chemiluminescence after the addition of a specific luminescent compound). This assay, by taking advantages of an automated chemiluminescence plate reader, overcomes SPA subjectivity of plaque counting, but shows some drawbacks, including a quite long experimental time (7 days) to obtain results. In addition both this method and SPA can be preferentially used for viruses able to induce CPE in cells.

On the contrary, our method allows a quicker detection of HSV-1 infectious foci (e.g., 24 h for HSV-1), does not need the use of cell overlay systems, requires a smaller volume of samples (12–120 μl in 96 well seeded cells – thus also taking advantage of multichannel pipettor use for handling large amount of samples), and provides an easily automated readout of the results by the aid of Odyssey instrumentation, thus increasing data reproducibility.

Differently to SPA, ICW requires an internal standard curve for each experiment. This may represent a limit. However, especially for basic research purposes, the use of internal HSV-1 standard allows to check the infectivity of the virus stock for each experiment and, at the same time, to easily compare experiments performed under different conditions (time p.i., m.o.i., etc).

Moreover, ICW recommends the use of a CellTag to normalize specific virus IF to cell number in the analyzed monolayer: this allows to overcome some technical troubles of SPA, especially those related to partial disruption of cell layer by high rate of virus infection and/or an high/diffuse and uncountable number of plaques (see for example **Figure 2C**). Similarly, fluorescence normalization allows ICW to easily detect foci of infection at the lowest sample concentration, up to single infected cells. At the same time, as reported by Weldon et al. (2010), NIR detection method provides a reduced autofluorescence background from biological molecules and limited light scatter than other visual IF assay, thus limiting potential bias in data interpretation and improving the assay sensitivity. By exploiting IMAGE-J software for avoiding unspecific fluorescence peaks, we further improve data analysis and interpretation of the results. On the other hand, ICW detection limit at the highest dilutions (e.g., 1:1000 dilution in **Figure 2B**) may be affected by the lower sample volume required by this assay with respect to SPA (50 μl vs. 300 μl), and by the different endpoints (24 h p.i. for ICW vs. 48–72 h p.i. for SPA). This means that in ICW a smaller number of viral particles are allowed to infect cells for a shorter time. In addition, the reagent sensitivity (especially primary antibodies against viral proteins) may play a role in virus detection. It is noteworthy to mention that ICW, by exploiting commercial antibodies that are able to recognize HSV-1 proteins, may be generally used for a wide range of virus strains. Eventual differences in protein expression among strains should not affect virus titre determination, since the method is based on an internal standard curve performed for each experiment with the same virus strain.

Beyond PRA, other methods have been so far developed for a rapid screening of antiherpetic compounds. They are mainly based on reporter systems such as luciferase and/or fluorescent proteins and are aimed at identifying antivirals targeting the early steps of HSV infection (Zhu et al., 2010; Matza-Porges et al., 2014; Maeda et al., 2016). These methods generally require the use of reporter-expressing recombinant viruses or cell lines transfected to express a reporter protein under a specific viral promoter. As we demonstrated for ACV (**Figure 3**), ICW and our modifications, by exploiting wild type HSV-1 strains and Vero cells, can be applied for the screening of antiviral drugs. In this view, the possibility to detect different viral proteins (e.g., gB and ICP4 as we demonstrated for HSV-1) makes ICW particularly suitable even for the characterization of different steps of viral replication and to explore the mechanisms of action of antiviral drugs, including those targeting early steps of the infection.

Overall, these advantageous features makes ICW a useful tool for virologists.

## Author Contributions

GDC and MEM designed the experiments. MF, DL, and MEM performed the experiments. MF, ATP, GDC, and MEM analyzed the data. MF, ATP, GDC, and MEM wrote the manuscript.

## Conflict of Interest Statement

The authors declare that the research was conducted in the absence of any commercial or financial relationships that could be construed as a potential conflict of interest.
