# Supplementary figures and images for: A Novel Method to Titrate Herpes Simplex Virus-1 (HSV-1) Using Laser-Based Scanning of Near-Infrared Fluorophores Conjugated Antibodies
Source: Front Microbiol. 2017 Jun 14;8:1085. doi: 10.3389/fmicb.2017.01085 (PMC5469900; doi:10.3389/fmicb.2017.01085)

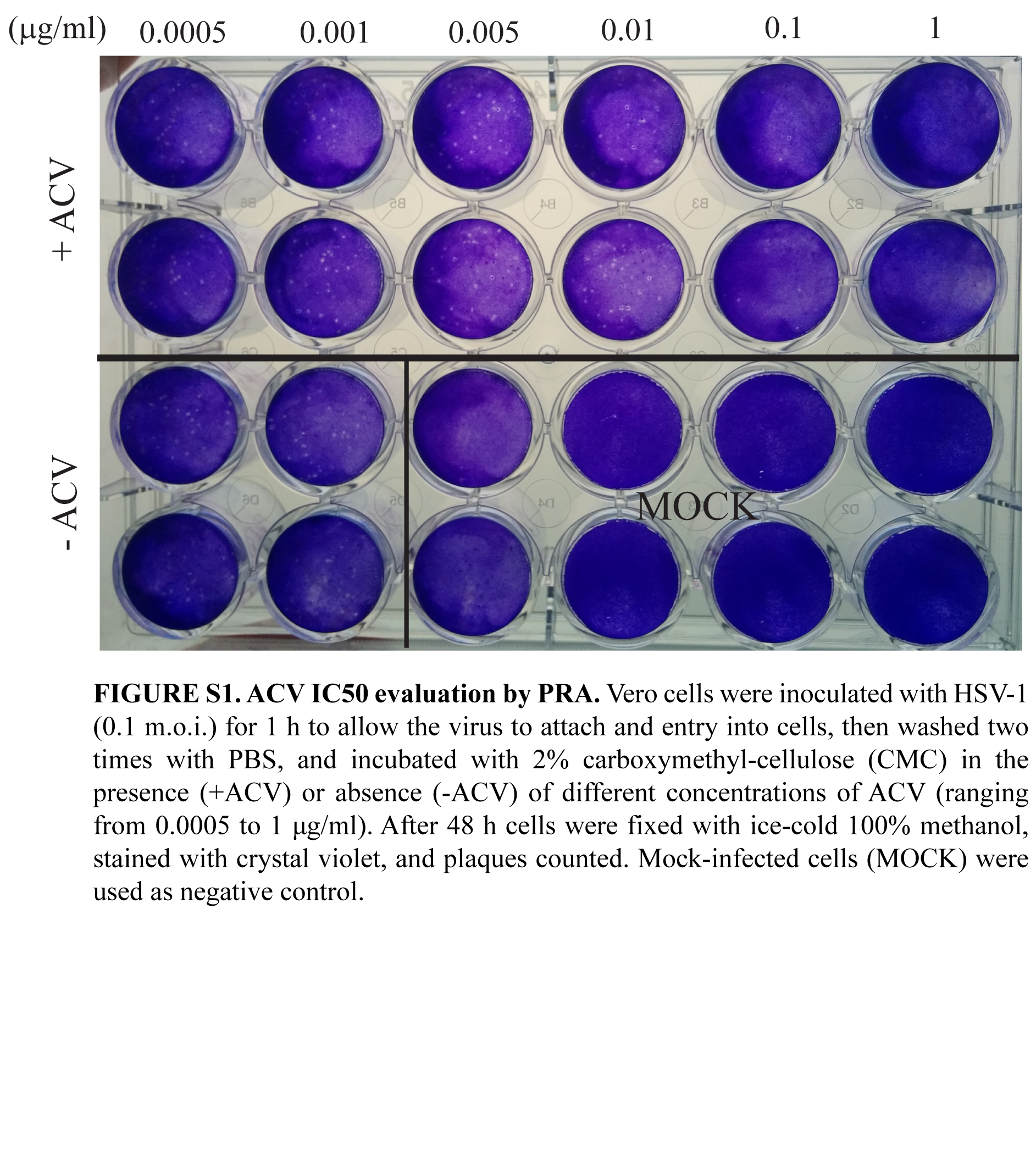

Supplement: Supplementary file 1 [file Image_1.tif]
